# PARSUC: A Parallel Subsampling-Based Method for Clustering Remote Sensing Big Data

**DOI:** 10.3390/s19153438

**Published:** 2019-08-05

**Authors:** Huiyu Xia, Wei Huang, Ning Li, Jianzhong Zhou, Dongying Zhang

**Affiliations:** 1 Yangtze River Waterway Bureau, Nanjing 210011, China; 2 School of Hydropower and Information Engineering, Huazhong University of Science and Technology, Wuhan 430074, China; 3 Yellow River Engineering Consulting Co., Ltd., Zhengzhou 450003, China

**Keywords:** clustering, parallel computing, remote sensing big data, MapReduce

## Abstract

Remote sensing big data (RSBD) is generally characterized by huge volumes, diversity, and high dimensionality. Mining hidden information from RSBD for different applications imposes significant computational challenges. Clustering is an important data mining technique widely used in processing and analyzing remote sensing imagery. However, conventional clustering algorithms are designed for relatively small datasets. When applied to problems with RSBD, they are, in general, too slow or inefficient for practical use. In this paper, we proposed a parallel subsampling-based clustering (PARSUC) method for improving the performance of RSBD clustering in terms of both efficiency and accuracy. PARSUC leverages a novel subsampling-based data partitioning (SubDP) method to realize three-step parallel clustering, effectively solving the notable performance bottleneck of the existing parallel clustering algorithms; that is, they must cope with numerous repeated calculations to get a reasonable result. Furthermore, we propose a centroid filtering algorithm (CFA) to eliminate subsampling errors and to guarantee the accuracy of the clustering results. PARSUC was implemented on a Hadoop platform by using the MapReduce parallel model. Experiments conducted on massive remote sensing imageries with different sizes showed that PARSUC (1) provided much better accuracy than conventional remote sensing clustering algorithms in handling larger image data; (2) achieved notable scalability with increased computing nodes added; and (3) spent much less time than the existing parallel clustering algorithm in handling RSBD.

## 1. Introduction

Geospatial data are one of the most significant types of big data, and the rapid growth of such data has imposed enormous challenges to current methodologies, applications, and infrastructures [1,2]. With the continuous improvement of earth observation satellite sensors and computer techniques, satellite remote sensing data has exploded in recent years, and a new research field called remote sensing big data (RSBD) has drawn great attention from academia and industry [3,4,5,6]. Mining hidden knowledge from RSBD for different applications, such as natural hazard monitoring, global climate change analysis, and urban planning, imposes significant computational challenges on scientists and researchers [7,8].

Clustering is an important data mining technique widely used in analyzing remote sensing data. Clustering can be defined as grouping a set of objects in such a way that objects in the same group are more similar to each other than to those in other groups [9]. Clustering is an effective technique for automatic remote sensing segmentation and classification since it does not require any training datasets in labeling classes for each pixel. Among the existing clustering methodologies, K-means [10], as well as its improved versions, such as iterative self-organizing data analysis (ISODATA) [11] and K-medoids [9], are most frequently used in remote sensing. However, conventional clustering algorithms are designed for relatively small datasets; when applied on problems with RSBD, they are generally too slow or inefficient for operation [12,13]. 

To deal with such issues, many efforts have been made to speed up clustering techniques for big data applications. The methods to speed up and scale up big data clustering algorithms are mainly in two categories: Single-machine clustering techniques and multi-machine clustering techniques [14,15]. In single-machine clustering techniques, a sampling-based method is commonly used to reduce the size of remote sensing data, which enables clustering algorithms to perform on a small sample of the input data instead of on the whole dataset. For example, Wang et al. proposed a density-based spatial clustering method (DBRS), using random sampling to reduce the running time for large-scale remote sensing datasets [16]. David et al. proposed a clustering method with sampling and subsampling strategies to cluster large datasets efficiently [17]. The classical bootstrap sampling technique was also investigated to speed up K-means clustering for RSBD [18]. In remote sensing applications, systematic sampling has been used to reduce the volume of the input images for ISODATA clustering [19]. However, sampling-based methods may not produce consistently reasonable results since not all members have an equal chance of being selected, which may lead to over or under estimation of the population [12]. Dimension reduction is another common method in single-machine clustering techniques to reduce the long execution time of RSBD clustering by projecting datasets from a high-dimensional space to a lower-dimensional space [20,21,22]. There is, however, still a tradeoff between the clustering speed and accuracy. Overall, the single-machine techniques have limited ability for speeding up clustering to handle RSBD.

On the other hand, the multi-machine clustering techniques are more popular due to their greater efficiency and scalability. Many big data clustering methods, based on parallel, distributed, and cloud computing frameworks, have been reported in the literature. For instance, Zhang et al. proposed MKmeans, a parallel K-means clustering algorithm with a message passing interface (MPI), which enables the clustering algorithm effectively in the parallel environment [23]. Xu et al. presented PDBSCAN, a parallel version of density-based spatial clustering of applications with noise (DBSCAN), one of the most widely used density-based clustering algorithms. The performance evaluation showed that PDBSCAN gained a linear speedup [24]. Recently, with the rise of cloud computing, MapReduce has become the most popular framework for big data clustering [25]. Zhao et al. proposed a parallel K-means clustering algorithm (PKmeans) based on MapReduce, which showed a good speedup [26]. Shahrivari et al. proposed a single-pass and linear time MapReduce-based K-means clustering method [27]. Kim et al. proposed a parallel density-based clustering algorithm called DBCURE-MR by using MapReduce [28].

In the remote sensing field, many researchers have been using parallel computing techniques to accelerate clustering for RSBD. Maulik and Sarkar proposed a parallel point symmetry-based K-means algorithm (ParSym) for image classification by using a distributed master-slave paradigm [29]. Based on ParSym, Du et al. proposed an improved parallel version called ParSymG, implemented by MPI [30]. Ye and Shi introduced a parallelizing ISODATA algorithm for unsupervised remote sensing imagery classification using a graphics processing unit (GPU, NVIDIA, Santa Clara, CA, USA) [31]. MapReduce-based clustering has also been investigated in remote sensing imagery. Li et al. proposed a parallel ISODATA clustering algorithm based on MapReduce to reduce the time cost by clustering algorithms when applied to a large number of images [32]. Parallel K-mans for clustering remote sensing images was reported by Lv et al. [33], which directly adopted the parallel strategy presented by [26]. Most of the existing parallel remote sensing clustering algorithms adopted a divide and conquer strategy to achieve parallelism [2]. The main idea is to partition the input image into several sub-images, assign them to different processors to compute independently, and then combine all the results into a final result. However, the divide and conquer strategy has considerable limitations since it brings numerous repeated calculations with input/output (I/O) operations [34,35]. The time cost is further challenged when executed on RSBD.

To overcome these limitations, in this paper, we propose a parallel subsampling-based clustering (PARSUC) method for RSBD. PARSUC employs a novel subsampling-based data partitioning method (SubDP) to realize three-step parallel clustering, and effectively avoids numerous iterative repeated calculations and I/O operations. Furthermore, a centroid filtering algorithm (CFA) is proposed in PARSUC to eliminate subsampling errors and guarantee the accuracy of the clustering result.

The main contributions of this paper are: (a) An efficient parallel clustering method for RSBD by using a novel data partitioning method (SubDP), (b) a centroid filtering algorithm (CFA) to eliminate the uncertainties and errors posed by subsampling, and (c) an implementation of PARSUC based on MapReduce.

The remainder of this paper is organized as follows. Section 2 describes the main steps and details of PARSUC. Section 3 introduces an implementation of PARSUC based on MapReduce. Section 4 evaluates the accuracy and time cost of PARSUC using RSBD. Section 5 discusses the experimental results and Section 6 concludes the paper with our future works.

## 2. Method

### 2.1. Traditional Parallelization Strategy

Most remote sensing algorithms have inherent parallelism. A straightforward approach is to divide the given remote sensing images into several partitions by a particular data partitioning method; run a parallel task on each partition concurrently and merge the results from all partitions. One operational partitioning method is the area-based method, which divides a scene of remote sensing imagery into equal-area sub-rectangles according to the abscissa and ordinate values. The area-based data partitioning methods are widely adopted by most of the existing parallel remote sensing clustering algorithms. However, such methods have some shortcomings when applied to RSBD. Each data partition obtained by the area-based partitioning method is a local area of the whole image, hence the clustering result from a partition represents only the local classification information. Simply merging the clustering results from different partitions leads to serious classification errors, since clustering needs the global information from the entire image. To solve this issue, existing parallel clustering algorithms generally use an iterative approach. They first execute clustering on each data partition in parallel to obtain local results, then they aggregate these local results to calculate a global conforming result by a specific function, such as voting or averaging. Next, the global conforming result is sent back to each parallel task as the input parameters for a new iteration of clustering on each partition. The process is repeated until the algorithm converges to an acceptable accuracy. However, this iterative approach suffers from serious performance bottlenecks since it requires numerous repeated calculations along with I/O operations on each data partition. The performance is further challenged when the approach is applied to large scale datasets, since clustering algorithms generally need much greater numbers of iterations to obtain convergence with increasing input size [36].

### 2.2. PARSUC

**Figure 1:**
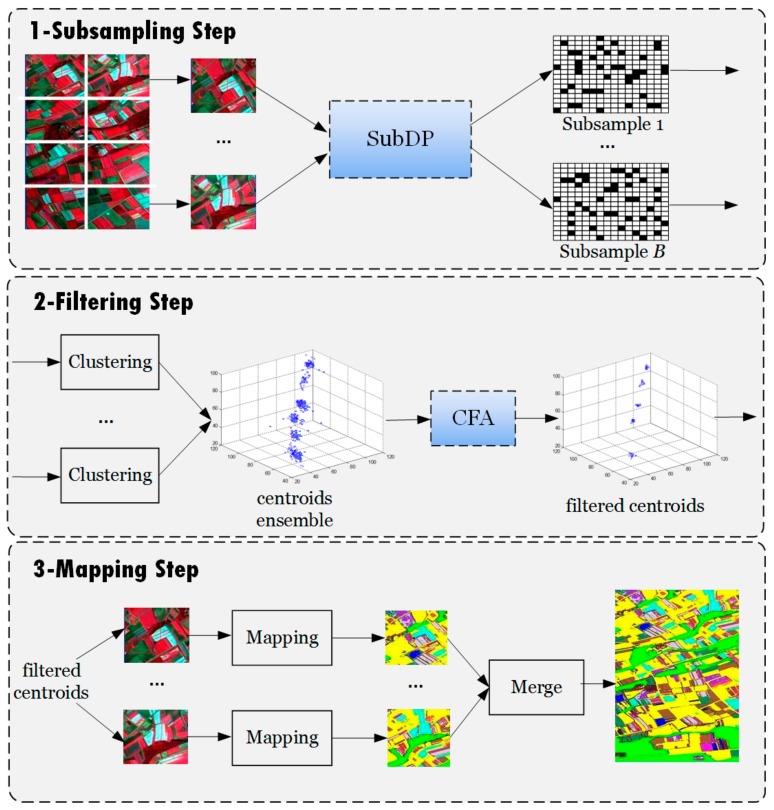
Framework of parallel subsampling-based clustering (PARSUC).

In this section, we propose a parallel subsampling-based clustering method (PARSUC) to deal with the abovementioned challenges. The framework of PARSUC is shown in Figure 1. PARSUC includes three steps: The subsampling step, the filtering step, and the mapping step.

#### 2.2.1. Subsampling Step

Instead of the area-based data partitioning method in the subsampling step, PARSUC adopted the subsampling-based data partitioning method (SubDP) to obtain parallel data sub-partitions. Subsampling is a classical statistical method by which a small and representative sample is taken from a large sample or population to reduce the data size. For remote sensing imagery subsampling, each single pixel was taken as the basic sampling unit. Given a scene of imagery with size *N*, subsampling at a rate of *ρ* meant drawing *Rounding* (*N* × *ρ*) pixels from the image in a random or systematic fashion. 

**Figure 2:**
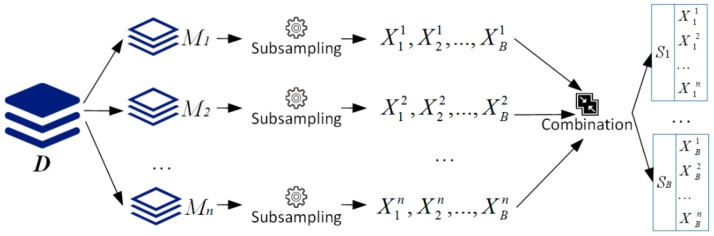
Subsampling-based data partitioning (SubDP) method.

Figure 2 illustrates the details of SubDP. In Figure 2, *D* indicates the input remote sensing image; *M*_1_ to *M_n_* are *n* data partitions obtained from *D* by using conventional area-based data partitioning methods. SubDP performed *B* rounds of subsampling at the rate of *ρ*∈(0,1) on each partition and obtained a set of intermediate subsamples: ***X_i_*** = {$X_{1}^{i},\ldots ,\;X_{B}^{i}$}, where *i* = 1 to *n*. The intermediate subsamples were then combined to form *B* subsamples according to their subscripts: ***S_j_*** = $X_{j}^{1}\cup X_{j}^{2}\ldots \cup X_{j}^{n}$, where *j* = 1 to *B*. Based on these *B* subsamples, the subsequent operations of PARSUC were designed.

Note that SubDP adopted a parallel approach to obtain subsamples rather than performing *B* rounds of subsampling directly on the original input image. This is owing to the following considerations: (1) Taking full advantage of parallel computing power for accelerating the subsampling process and (2) guaranteeing that the selected pixel points are evenly distributed in the whole space of the input image.

Although SubDP still used the parallel data partitions from the conventional area-based partitioning method, the design ideas were totally different. Each data partition from the conventional area-based partitioning method had only local information of the image, while the data partition from SubDP preserved the global pixel value distribution information. Moreover, the subsampling rate and the number of subsamples, which had a direct impact on the total computational load, were controllable. These key characteristics of SubDP provided PARSUC with a flexible and non-iterative approach to achieve parallelism. However, the correct choice of the subsampling rate *ρ* and the number of subsamples *B* was also closely related to the accuracy of the algorithm, because a subsample generally had better representation with more sample pixels and more subsamples may cause lower information loss. We will discuss the impact of these two parameters in Section 4, based on the experiments.

#### 2.2.2. Filtering Step

In the filtering step, PARSUC first executed a clustering algorithm on *B* subsamples in parallel. This clustering algorithm is not restricted to a specific algorithm and can be a variation of centroid-based clustering algorithms, such as K-means, ISODATA, K-means++, or K-medoids. One of the main advantages of PARSUC is that it can be used as a general framework for several common clustering algorithms, since no single clustering algorithm can classify all different sources of remote sensing imageries successfully.

After clustering was applied to each subsample, there were *B* groups of output: *P* = {*P*_1_, …, *P_B_*}. Each component *P_j_* in *P* was a set of clusters: $P_{j}=\left\{C_{1}^{j},C_{2}^{j},\;\ldots ,\;C_{K\left(j\right)}^{j}\right\}$, where *j* = 1 to *B* and *K*(*j*) denotes the number of clusters from the *j*th subsample. To find the final target clustering of the original image from the intermediate clustering results *P*, PARSUC built a consensus function based on cluster centroids. In clustering analysis, a centroid refers to the most representative point within a cluster, usually taking the mean value of all the instances in the cluster. For a given set of clusters $P_{j}=\left\{C_{1}^{j},C_{2}^{j},\;\ldots ,\;C_{K\left(j\right)}^{j}\right\}$, the centroid of each cluster is defined as Equation (1):

(1) $$v_{i}^{j}=N{\left(C_{i}^{j}\right)}^{-1}\overset{N(C_{i}^{j})}{\underset{m=1}{\sum }}x_{m}$$

where *i* = 1 to *K*(*j*) and $N\left(C_{i}^{j}\right)$ denotes the number of instances in cluster $C_{i}^{j}$.

PARSUC gathered all the centroids drawing from *P* into an ensemble *V* = {*V*_1_, …, *V_B_*}. Each component *V_j_* in *V* was a set of centroids: $V_{j}=\left\{v_{1}^{j},v_{2}^{j},\ldots ,v_{K\left(j\right)}^{j}\right\}$, where *j* = 1 to *B*. In case of remote sensing clustering, each centroid is a *d*-dimentional vector, where *d* is the number of bands in the input image. As mentioned above, subsamples preserved the pixel value distribution information of the same original image; thus, the statistical properties of different subsamples were similar to each other. Consequently, the centroids obtained from these subsamples were supposed to be spatially close to each other and were supposed to form several separated compact groups in the *d*-dimensional space. Figure 1 takes a three-dimensional coordinate system as the example to show the way centroids are grouped. However, because of the randomness of subsampling and the consequent information loss, the centroids obtained from those subsamples with low representation of the population may have been distant from the rest. These centroids should be filtered as outliers since they seriously affected the accuracy of the final clustering result.

**Figure 3:**
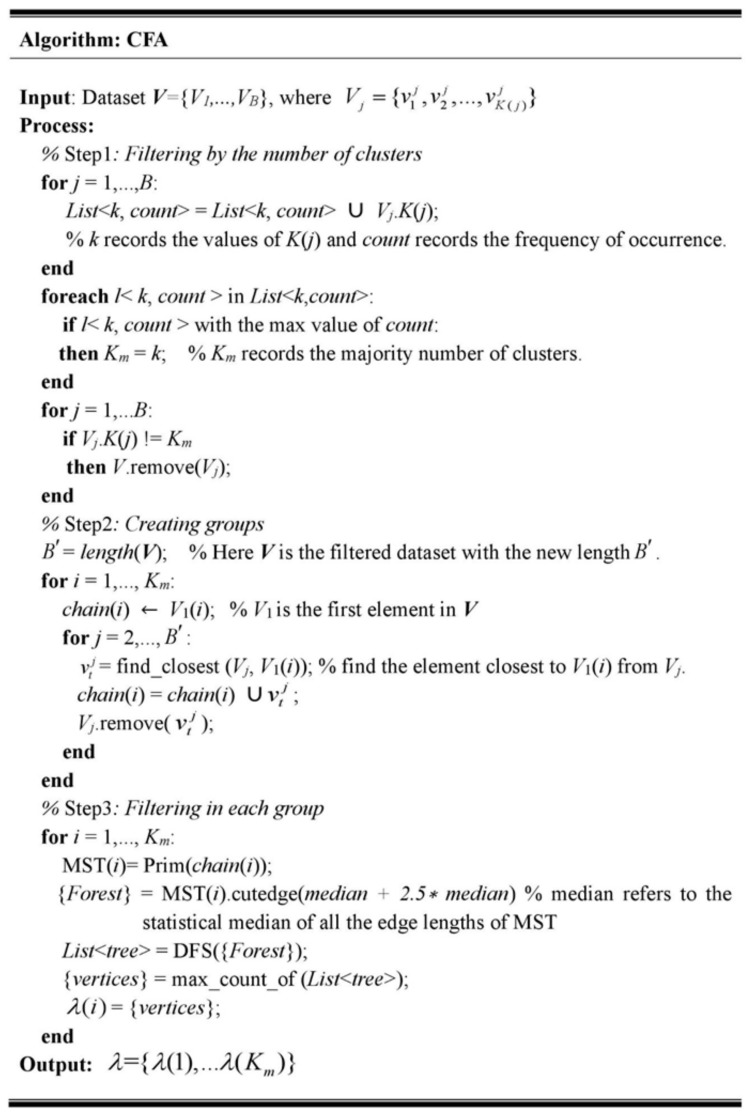
Pseudocode of the centroid filtering algorithm (CFA).

We present a centroid filtering algorithm (CFA) to filter these “bad” centroids from the ensemble to reach a global consensus of the position of the centroids in the target clustering. The pseudocode of CFA is shown in Figure 3.

As shown in Figure 3, CFA mainly included the following three steps:

1. Filtering by the number of clusters.

In this step, the number of clusters was considered the first filtering condition for those algorithms such as ISODATA, whose number of clusters usually dynamically changes in each iteration. CFA first counted all different *K*(*j*) of *V_j_* from *V* and recorded them in a list. Secondly, it calculated the most frequent value (denoted by *K_m_*) in the list. Next, CFA removed all *V_j_* values whose *k* were not equal to *K_m_* from *V.* It should be noted that, for clustering algorithms with a determined number of clusters, such as K-means, this step could have been skipped since all outputs had the same number of clusters.

For example, suppose that there were six elements in *V* = {*V*_1_, *V*_2_, *V*_3_, *V*_4_, *V*_5_, *V*_6_} and their number of clusters were recorded in the list = <3, 5, 5, 6, 5, 5>. The most frequent value in the list is *K_m_* = 5. In the first filtering step, two elements *V*_1_ and *V*_4_ were removed from *V*. The removal rate in this case was 33.3 percent. 

In practice, we found that the removal rate varied from parameters of SubDP. Table 1 shows the distribution and variation of the removal rate with a different number of subsamples *B* and subsampling rates *ρ*, and the results were averaged on 10 different remote sensing images. As shown in Table 1, the removal rates were generally larger with a lower subsampling rate. When subsampling rates were equal to 1 percent, nearly half of the elements were removed from *V*. This indicates that there were great differences between the subsamples and the cluttering, results from which can vary considerably. When the subsampling rate increased from 20 to 30%, the removal rates decreased significantly, which meant that most elements in *V* had the same number of clusters. The results also indicated that the removal rate had little relationship with the number of subsamples *B*.

2. Creating groups.

As mentioned above, similar centroids naturally formed several separate compact groups in the *d*-dimensional space. However, we could not find any correspondence between centroids and groups because centroids have no labels. To identify which centroids belonged to the same group, we organized the unlabeled centroids by their similarity to create groups. We employed the classical method consensus chain to achieve this goal [37]. In the consensus chain, the similarity between two centroids was defined as the Euclidean distance between their values. As illustrated in Figure 3, CFA traverses all elements from the filtered dataset *V* then adds the closest centroids to the same chain by calculating their Euclidean distances. After all *K_m_* chains were established, the centroids in the same chain constituted a group.

3. Filtering in each group.

In this step, CFA first built a minimum spanning tree (MST) from a weighted undirected graph for each created group by using Prim’s algorithm [38]. The vertices of a MST were the centroids in the same group, and the weight of the edges were the Euclidean distances between the centroids. Next, CFA removed the long edges of the MST by a user-defined threshold to cut off the connections between outliers and those compact groups. After cutting the long edges, the MST converted to a forest. Finally, CFA selected the vertices of the largest connected tree from this forest, which was found by depth-first searching, and discarded the rest as outliers.

**Figure 4:**
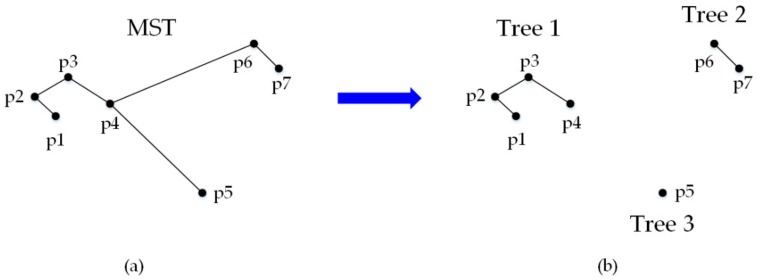
The minimum spanning tree (MST) (**a**) converting to a forest (**b**).

Figure 4 illustrates how a MST converts to a forest by an example of two-dimensional points. Each point represents a centroid. Figure 4a shows an example of buiding a MST from centroids, and Figure 4b shows the resulting forest after cutting long edges (p4p6 and p4p5) from such a MST. There were three trees in the forest, the largest connected Tree1 (p1, p2, p3, and p4) was then selected. Tree2 (p6 and p7) and Tree3 (p5) were discarded as outliers.

The number of trees in the forest and the number of vertices of each tree was directly determined by the threshold for cutting long edges. If the threshold was set too high, the algorithm could not find any long edges to cut. Outliers could not be distinguished since there was only one tree in the forest. If the threshold was set too low, most edges of the MST were cut. Outlierscould also not be effectively distinguished since there were lots of scattered trees in the forest and most of them had only one vertice. It was difficult to present a stable threshold without prior knowledge about the distribution of outliers in each group, however, we leveraged the statistical median at all edge lengths to achieve that goal. In practice, we set the threshold as 2 × *median*, 3 × *median*, 4 × *median*, and 5 × *median*, respectively.

To evaluate the rationality of the threshold, we set an index *I_threshold_* to denote the ratio of the number of vertices of the largest tree to the total number of vertices of the MST. For example, in Figure 4, the *I_threshold_* was equal to 4/7. Essentially, the vertices of the largest tree represent the largest group of points that were spatially closest to each other. Ideally, this ratio should be in the 50%~70% range since, in most cases, filtering 30–50% points as outliers was enough for finding the most compact group. Table 2 shows the variation of *I_threshold_* with different thresholds. The statistical results from multiple tests on different remote sensing images indicated that it was reasonable to set the threshold from 3 × *median* to 4 × *median*. Although the median-based method for threshold determination can meet the current requirements of applications, it has much room for improvement. We will investigate other statistical-based methods to further improve the performance.

#### 2.2.3. Mapping Step

**Figure 5:**
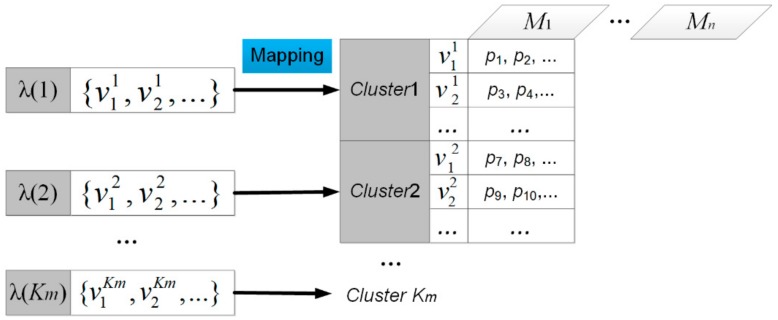
The mapping operation of PARSUC.

After the filtering step, we obtained *K_m_* groups {*λ*(1), …, *λ*(*K_m_*)}. Each group contained a number of filtered centroids that reached a consensus with the final clustering result. In the mapping step, PARSUC produced a final clustering result through these groups of filtered centroids by a mapping operation. Figure 5 illustrates how mapping works. For example, $v_{1}^{1}$ and $v_{2}^{1}$ denote the centroids in group *λ*(1), *M*_1_, …, *M_n_* denote data partitions of the input image, and *p*_1_ and *p*_2_ denote pixels from *M*_1_. In the mapping operation, PARSUC calculated the Euclidean distance between pixels and centroids, then assigned the pixels to their closest centroid. In Figure 5, *p*_1_ and *p*_2_ were assigned to $v_{1}^{1}$, and *p*_3_ and *p*_4_ were assigned to $v_{2}^{1}$. Next, PARSUC categorized each assigned pixel according to its centroid’s group number. For example, *p*_1_, *p*_2_, *p*_3_, and *p*_4_ were categorized to Cluster1, and *p*_7_, *p*_8_, *p*_9_, and *p*_10_ were categorized to Cluster2. After the mapping process, PARSUC aggregated all of the clustered pixels from different data partitions and merged them into the final clustering result of the original input image.

## 3. Implementation

**Figure 6:**
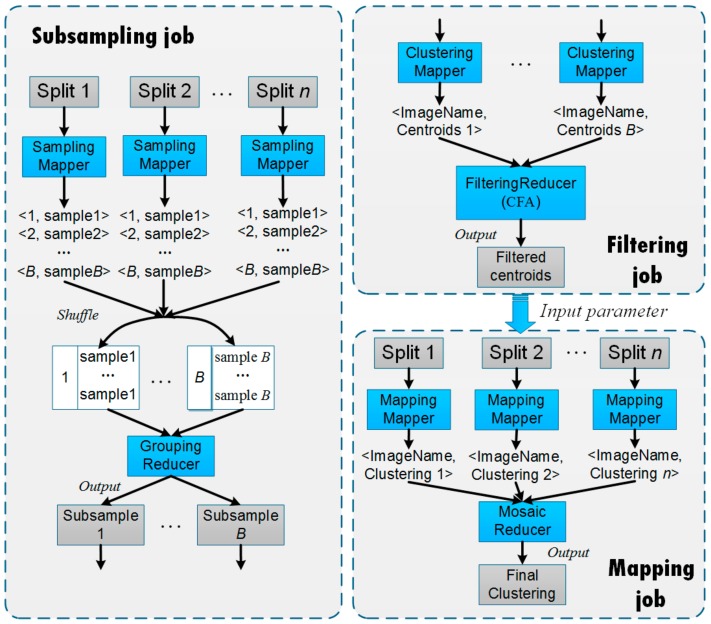
The MapReduce-based implementation of PARSUC.

In this section, we present an implementation for PARSUC based on the MapReduce model. Note that there are multiple traditional parallel programming models that could be used to implement PARSUC, such as MPI or compute unified device architecture (CUDA). We chose MapReduce due to its high scalability, reliability, and fault tolerance. In fact, MapReduce is now the most commonly-used tool in cloud computing for handling big data. As shown in Figure 6, the MapReduce-based implementation of PARSUC is composed of three different MapReduce jobs: The subsampling job, the filtering job, and the mapping job.

The subsampling job is the implementation of SubDP. The subsampling job is composed of the SamplingMapper and GroupingReducer. The input image is first partitioned into *n* partitions (called splits in MapReduce) using the area-based data partitioning method. Each SamplingMapper takes a split as the input and performs *B* rounds of subsampling at a user-defined sampling rate. The outputs of SamplingMappers are *B <Key, Value>* pairs, where *Value* stores all the sampling results with their *Key* index, ranging from 1 to *B*. In the shuffle operation, MapReduce reorganizes the *B* pairs based on their *Key*s, such that all the pairs belonging to the same *Key* are assigned to the same GroupingReducer. The GroupingReducer then merges them to form *B* subsamples.

The filtering job is composed of the ClusteringMapper and FilteringReducer. Each ClusteringMapper reads a subsample, applies a user-defined clustering algorithm, and extracts the centroids from the resultant clusters. The output of each ClusteringMapper is a <*Key*, *Value*> pair, where the *Value* stores the centroids and the *Key* is an identical value, e.g., image name, to transfer all the output <*Key*, *Value*> pairs to the same FilteringReducer. The main function of the FilteringReducer is to gather all the centroids from the ClusteringMappers, perform CFA over them, and output the filtered centroids.

The mapping job is composed of the MappingMapper and MosaicReducer. The MappingMapper takes the filtered centroids from the filtering job as the input parameters and executes the mapping operation on each split. Once the MappingMapper is complete, the MosaicReducer collects the intermediate clustering results and then merges them by their coordinate values into the final clustering result.

## 4. Results

Experiments were conducted to evaluate PARSUC in terms of both accuracy and time cost. PARSUC was implemented by MapReduce and deployed on a Hadoop cluster, which was built on a private cloud platform. The private cloud consists of 10 servers, and each server was equipped with Intel Core i5 3.1GHz CPU and 16GB RAM, and a VMware workstation was used to provide virtual machine instances. Hadoop YARN 2.2.0 was installed with default configurations, including one master node and 20 slave nodes. Hadoop 2.6.0 was installed on each node and the operating system was Ubuntu 14.04.

### 4.1. Accuracy Validation

In this study, we use a sum of squared error (SSE) to verify the accuracy of PARSUC. A SSE is a common partitioned clustering criteria, aiming to measure the squared errors between each data point and the cluster center to which the data point belongs to. A smaller SSE means a more accurate clustering result. The SSE is defined as Equation (2):

(2) $$SSE=\overset{K}{\underset{k=1}{\sum }}\overset{n_{k}}{\underset{j=1}{\sum }}||x_{j}-v_{k}||{}^{2}$$

where *K* is the number of clusters, *n_k_* is the number of points in the *k*th cluster, and $||x_{j}-v_{k}{||}^{2}$ represents the squared Euclidean distance between *x_j_* and its corresponding centroid, *v_k_*.

**Figure 7:**
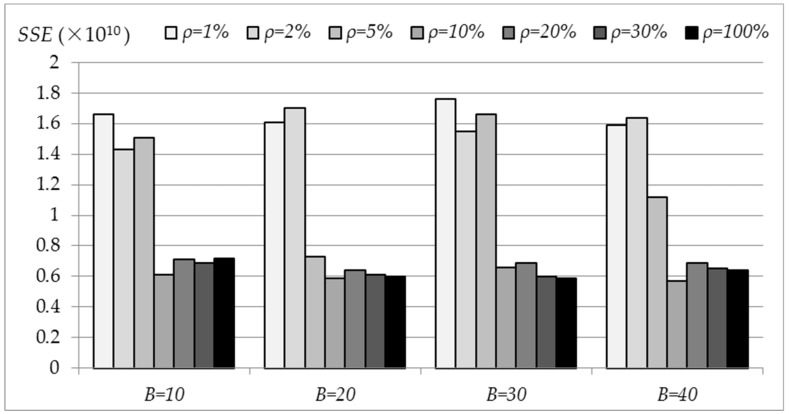
Variation of sum of squared errors (SSEs) for different combinations of *ρ* and *B*.

As discussed in Section 2.2.1, the subsampling rate, *ρ*, and the number of subsamples, *B*, are two key parameters in subsampling that closely relate to the accuracy of PARSUC. In our first round of experiments, we investigated how these two parameters impacted the accuracy of the results. Remote sensing imageries involved in the experiments included 20 scenes of Chinese GaoFen-1 (GF-1) high-resolution images with four bands (blue, green, red, and near infrared) at 8-meter spatial resolution. The two parameters were alternated among all runs, the *ρ* taking values of 1%, 2%, 5%, 10%, 20%, and 30%, and the *B* taking values of 10%, 20%, 30%, and 40%. PARSUC was executed on a total of 20 scenes of GF-1 imageries and the mean values of SSEs of each scene were calculated. Figure 7 shows the variation of SSEs for different combinations of *ρ* and *B*. The horizontal axis represents the value of *B*, ranging between 10 to 40% with a step size of 10%, and the vertical axis represents the value of SSE (×10^10^).

Figure 7 illustrates that no matter how *B* changes, SSE values are generally much larger with a lower subsampling rate, *ρ*. Specifically, when *ρ* = 1% and 2%, the SSE are in the 1.42 × 10^10^ ~ 1.75 × 10^10^ range. When *ρ* takes higher values from 5% to 30%, the SSE drops to the 0.58 × 10^10^ ~ 1.15 × 10^10^ range. This observation indicates that the accuracy of PARSUC is more sensitive to the variation of the subsampling rate than to the number of subsamples. A higher subsampling rate can improve the clustering accuracy. However, this improvement is limited. As shown in Figure 7, SSE did not show any distinct tendency to decrease when *ρ* rose from 10% to 30%, instead it just fluctuated within a certain range. The findings held even when *ρ* reached 100%. That was mainly because, as the subsampling rate increased to a certain value, multiple subsamples had already contained enough detailed information for clustering, hence the accuracy of PARSUC no longer increased.

We started *B* from 10 rather than one, because when *B* = 1, there would be only one subsample drawn from the original data. The accuracy of PARSUC will entirely depend on the quality of one sampling process. The results from each test may differ greatly even on the same dataset. In order to obtain stable and comparable results, more than 10 subsamples were used in PARSUC. However, the selection of *B* and its impacts on the stability of the results will be investigated in our future work.

In the next step, we evaluated the clustering accuracy by comparing PARSUC with two conventional remote sensing clustering algorithms, K-means and ISODATA. As mentioned above, in the filtering step of PARSUC, the clustering algorithm is user-defined. For comparison purposes, we implemented two versions of PARSUC: K-means and iterative self-organizing data analysis (ISODATA), which were used in implementing the ClusteringMapper. We call them PARSUC(KM) and PARSUC(ISO). The initial number of clusters was set to 10 and the maximum number of iterations was set to 20. The subsampling rate, *ρ,* of PARSUC was set to 10%, and the number of subsamples, *B,* was set to 10%. The conventional K-means and ISODATA and PARSUC(KM) and PARSUC(ISO) were performed on GF-1 imageries with different sizes, then SSEs were calculated.

Table 3 shows the comparison results of the clustering accuracy between PARSUC(KM) and K-means, and Table 4 shows the comparison results of the clustering accuracy between PARSUC(ISO) and ISODATA. Table 1 illustrates that comparing with the conventional K-means, PARSUC(KM) has no significant advantages in accuracy when processing small sizes of imageries. However, as the image size increases, PARSUC(KM) becomes more accurate than K-means. When the image size reached 4000 × 4000, the SSE of PARSUC(KM) was only approximately 24% that of K-means. Similarly, as shown in Table 4, PARSUC(ISO) performed much better than conventional ISODATA in accuracy when processing larger sizes of imageries.

### 4.2. Accuracy Validation by Two Cases

Except for the validation of SSE, we further testified the accuracy of PARSUC by two application cases compared to K-means and ISODATA. In these cases, half of the ground truth samples were used to label each cluster with a specific land cover type. The remaining samples were performed to validate the accuracy of different clustering algorithms. Note that the results of the clustering algorithms didn’t have class labels. To validate the clustering results with 50% of ground truth samples and to calculate classification accuracy, we first manually labeled each cluster with a type of land cover, according to the spatial distribution correspondence between clustering results and the other 50% of ground truth samples.

Case1: Croplands Classification.

**Figure 8:**
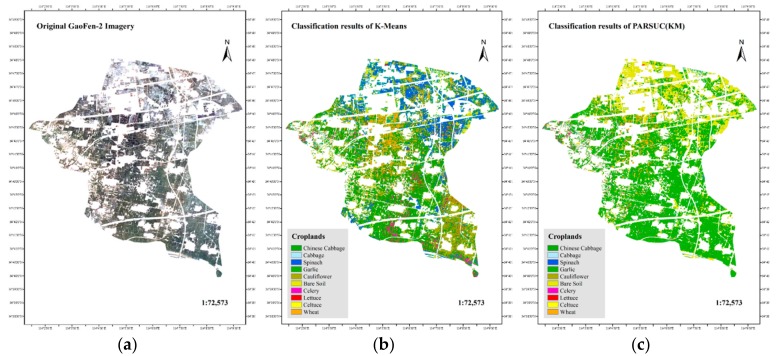
Original GaoFen-2 imagery (**a**), classification results of PARSUC(KM) (**b**) and K-means (**c**).

One scene of a Chinese GaoFen-2 remote sensing image (one panchromatic band at 1 meter resolution and four multispectral bands at 4 meter resolution) captured on 27 March, 2018 was used to classify the crop type in Guandu Town, Zhongmou County, Zhengzhou City, Henan Province, China. First, we fused the panchromatic band and four multispectral bands, and the size was 16,637 × 20,930 pixels per fused band. Additionally, we masked the fused imagery with the vector files of Guandu Town boundary, road, and building and reserved only croplands, including Chinese cabbage, cabbage, spinach, garlic, cauliflower, bare soil, celery, lettuce, celtuce, and wheat. A total of 10 types of crop differed in spectral specifications and textural features. PARSUC(KM) and K-means were executed on the masked imagery. The initial number of clusters was set to 10, and the maximum number of iterations was set to 20. The subsampling rate of PARSUC(KM) was set to 10%, and the number of subsamples was set to 10%. Figure 8a shows the original GaoFen-2 imagery used in this study with true color. Figure 8b is the classification result of K-means, and 8c refers to the classification result of PARSUC(KM).

A total of 179 in situ measured cropland samples on 20 March, 2018 were performed to validate the classification results by means of Kappa coefficient and overall accuracy with a confused matrix. The overall accuracy and Kappa coefficient of PARSUC(KM) results (62.8976%, 0.5229) were higher than those of K-means results (47.2569%, 0.4126).

**Figure 9:**
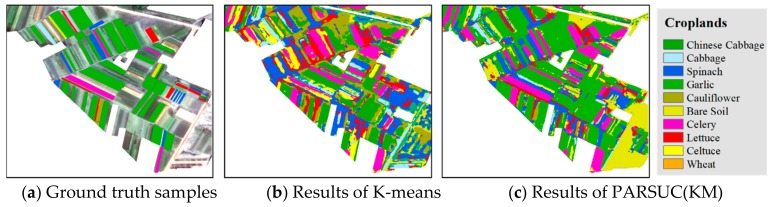
Ground truth samples, classification results of PARSUC(KM) and K-means at region 1.

**Figure 10:**
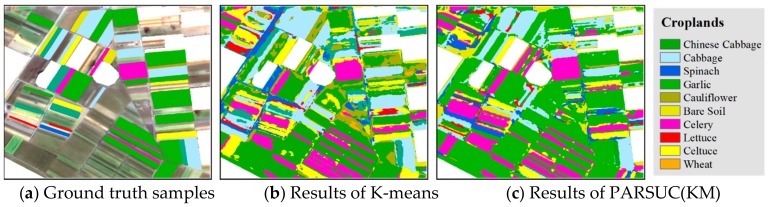
Ground truth samples, classification results of PARSUC(KM) and K-means at region 2.

To more specifically compare ground truth samples, classification results of PARSUC(KM), and K-means we selected two regions of classification results in Figure 8b,c and enlarged them, as shown in Figure 9 and Figure 10. 

In region 1, Figure 9 showed PARSUC(KM) identified crop fields more completely than K-means in the majority of crop types. Take the recognition of Chinese cabbage fields as an example. PARSUC(KM) identified 25 of all 27, while K-means captured only 19 of 27 Chinese cabbage fields. The commission and omission probability of PARSUC(KM) result was less than that of the K-means result. PARSUC incorrectly interpreted of three lettuce fields and one cauliflower field at a small size. Region 2 showed similar patterns as region 1. Figure 10 demonstrates that PARSUC(KM) matched 15 Chinese cabbage fields of 15 and 8 cabbage fields of 9 ground truth samples completely, while K-means mistook cauliflower as Chinese cabbage fields. 

Case2: Water Body Extraction

**Figure 11:**
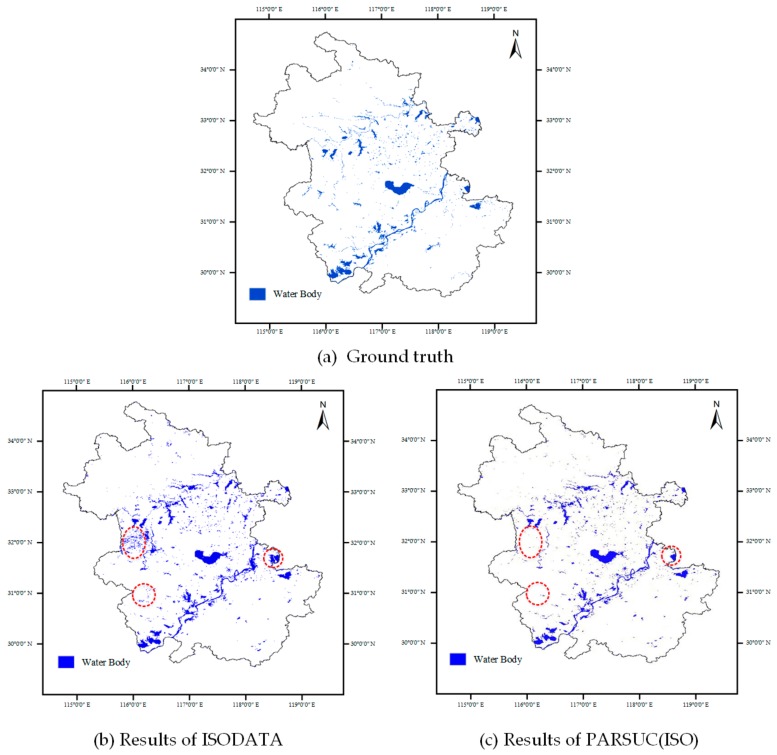
Ground truth, water body extraction results of ISODATA and PARSUC(ISO).

Another comparison experiment was performed on the time series of normalized difference vegetation index (NDVI) raster datasets, which were derived from monthly moderate resolution imaging spectroradiometer (MODIS) sensor imagery with 250-meter resolution in 2015. The vegetation indices were retrieved from the MODIS surface reflectance of a red waveband (band 1) and near-infrared band (band 2). Clustering on the time series of the NDVI is an effective approach for retrieving water bodies since their NDVI values are quite stable within the whole year. The traditional ISODATA and PARSUC(ISO), both with the initial number of clusters (10), were performed on the 12-layer NDVI time series raster datasets, and then the resultant classes of water bodies were extracted manually. Figure 11 shows the comparison results between ISODATA and PARSUC(ISO). Figure 11a is the ground truth of water bodies from the global land cover, 11b is the ISODATA result, and 11c is the PARSUC(ISO) result. As depicted in Figure 11b,c, the main water bodies in Anhui province of China can be detected by clustering algorithms. However, plenty of scattered areas were mistakenly recognized as water bodies by the traditional ISODATA algorithm (red circles). In comparison, PARSUC(ISO) had much better performance in recognizing the water bodies.

### 4.3. Time Cost

Time cost and scalability of PARSUC were also examined. A total of 150 scenes of remote sensing imageries were used for evaluating the time cost of PARSUC, including 50 scenes of GF-1 images at three different sizes: 5000 × 5000, 10,000 × 10,000, and 20,000 × 20,000. In our configuration, one mapper job was assigned to each slave node for establishing correspondence between each mapper and node. PARSUC was executed five times on a total of 150 scenes of imageries and the average running times were recorded. For each imagery size, Hadoop slave nodes were increased from 5 to 20 with a step of 5. 

**Figure 12:**
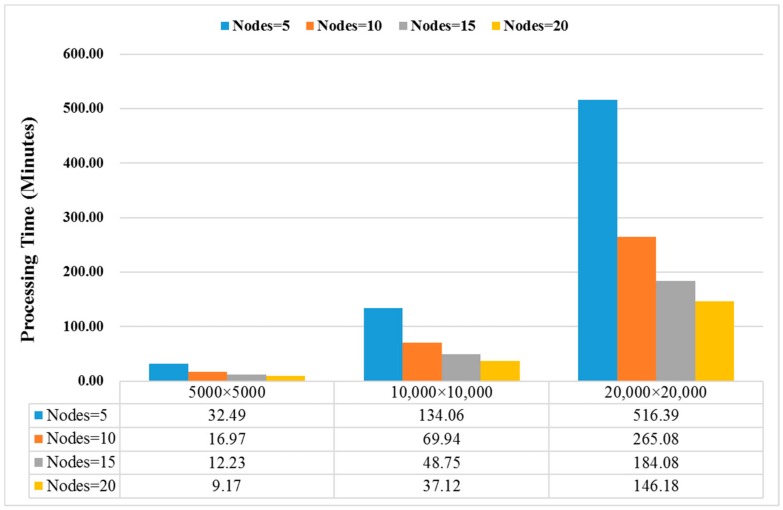
Processing time of PARSUC with different nodes and image sizes.

Figure 12 shows the computation time (in minutes) of PARSUC for processing three different sizes of remote sensing images, as the number of Hadoop slave nodes increased from 5 to 20. Overall, the processing time of PARSUC decreased dramatically when more slave nodes were utilized. Specifically, when processing images with size 5000 × 5000, the average processing time was sharply reduced from 32.49 min to 9.17 min as the nodes increased from 5 to 20. When processing larger images with size 20,000 × 20,000, the time consumption was reduced from 516.39 min to 146.18 min, an almost fourfold reduction.

**Figure 13:**
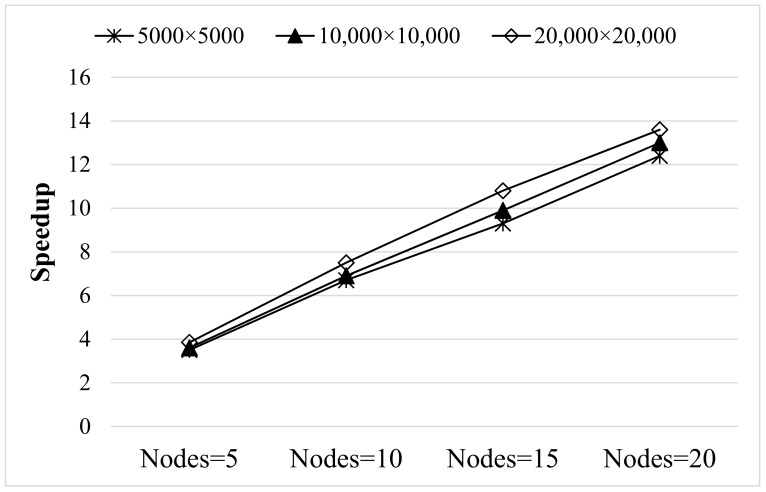
Speedup of PARSUC with different number of nodes.

Figure 13 shows the speedup that PARSUC achieved with an increased number of nodes. PARSUC obtained linear speedup with respect to an increase in the number of slave nodes. We also observe that the larger size of imageries attained higher speedup than smaller ones. Note that this scalability can be achieved by simply configuring and adding new slave nodes to the Hadoop cluster, without any changes to the PARSUC code.

To demonstrate the advantage of PARSUC over the existing parallel remote sensing clustering algorithms in time cost, further experiments were conducted by comparing PARSUC with a typical MapReduce-based parallel clustering algorithm proposed by Li et al. [32]. We implemented their algorithm in the same development environment as PARSUC, hereinafter named as MapReduce-based parallel clustering (MPC). MPC was also executed five times on a total of 150 scenes of GF-1 imageries with different sizes, and the average running times were recorded.

Table 5 demonstrates the comparison results of the time cost (in minutes) between PARSUC and MPC in handling the same scale of test remote sensing imageries. We can see that the time cost of both PARSUC and MPC improve as the number of slave nodes increases. However, under the same computation environments, the processing time of PARSUC is far less than MPC in scope with the same size of imageries. We analyze the reason later in the discussion.

## 5. Discussion

In investigating the influences of the subsampling rate and the number of subsamples on results of PARSUC, we find that, although the accuracy of PARSUC can be improved by increasing the subsampling rate, this improvement eliminates when the subsampling rate reaches a certain threshold. This is because subsamples with a low subsampling rate cannot carry enough information of the original image for clustering and may cause significant errors. Increasing the subsampling rate to a suitable level will cause significant improvements in accuracy. However, PARSUC adopts multiple subsamples to compensate for the shortage of the individual subsamples. As the subsampling rate increases to a certain value, multiple subsamples have already contained enough detailed information for clustering; therefore, the accuracy of PARSUC no longer increases. Informed by this observation, we can determine a relatively low subsampling rate, e.g., 10 to 20%, in getting parallel subsamples to reduce the total amount of computation for processing RSBD. Another important observation is that the number of subsamples has little impact on the clustering accuracy. Therefore, in an operational application, we can choose a relatively small number of subsamples to further reduce the total computational burden. The recommended number of subsamples in this study is 10.

In comparison with the most widely used remote sensing clustering algorithms, K-means and ISODATA, PARSUC shows a significant advantage in accuracy when dealing with a larger size of imageries. These promising results can be explained; for big datasets, conventional clustering algorithms do not converge to an acceptable accuracy within finite iterations. Moreover, the accuracy of conventional clustering algorithms suffers from the drawbacks of local minima and inefficient clustering initializations, and these drawbacks are magnified in processing RSBD. However, PARSUC overcomes these shortcomings by aggregating multiple clusterings to obtain more stable and accurate results. Although defective clustering may still exist in subsamples, they can be detected effectively and eliminated by our proposed CFA.

PARSUC demonstrates notable speedup in our experiments, especially when processing RSBD. Benefiting from the scalability and flexibility features of MapReduce, the computation capacity of PARSUC can flexibly scale up to meet the rapid growth in processing requirements. Furthermore, this scalability can be achieved by simply configuring and adding new computing nodes to the cluster without any changes to the code. 

Compared to the existing MapReduce-based parallel clustering algorithm, MPC, PARSUC spent much less time in processing the same test remote sensing imageries in the same computation environment. This is mainly because MPC uses iterative MapReduce jobs to obtain the optimum clustering results. During each iteration of MPC, the entire scenes of imageries must be read and written to disks, costing a large number of I/O operations. However, PARSUC achieves that goal by using only three different MapReduce jobs, among which only the first and third jobs have to load the entire imagery dataset. Therefore, PARSUC performs much more effectively than MPC in dealing with RSBD.

## 6. Conclusions

In this paper, we proposed a parallel clustering method, PARSUC, for improving the performance of RSBD clustering in terms of both efficiency and accuracy. PARSUC leveraged a novel subsampling-based data partitioning method, SubDP, to realize three-step parallel clustering, effectively solving the notable performance bottleneck of existing parallel clustering algorithms; that is, they must cope with numerous repeated calculations to obtain a reasonable result. Furthermore, PARSUC adopted the centroid filtering algorithm, CFA, to eliminate the subsampling errors and to guarantee the accuracy of the clustering results. PARSUC was implemented on a Hadoop platform by using the MapReduce parallel model. Experiments performed on RSBD with different sizes showed very promising results. First, in dealing with a larger scale of remote sensing imageries, PARSUC performed much more accurately than conventional remote sensing clustering algorithms, K-means, and ISODATA. Second, PARSUC achieved notable scalability with the addition of more computing nodes. Third, compared to the existing MapReduce-based parallel clustering method, PARSUC demonstrated less time cost in handling RSBD. Another important feature of PARSUC was that the total amount of computation required could be flexibly controlled by adjusting the subsampling parameters. The experimental results indicated that PARSUC obtained acceptable results at very low subsampling rates and numbers of subsamples. With these features, PARSUC is operational to analyze RSBD for accurate and stable clustering results. 

Although SubDP shows promise for efficient clustering of RSBD, other data partitioning methods based on statistical sampling techniques will be investigated to further improve the performance of PARSUC. Meanwhile, the outlier detection method used in CFA still has room for improvement in future works, and the need to improve that method is an open problem in geo-information research. In this version of PARSUC, only centroid-based clustering algorithms are supported, and we will continue to improve PARSUC to support more types of clustering algorithms, such as DBSCAN.

## Figures and Tables

**Table 1:** Removal rate (in %) of the first filtering step of CFA.

	*ρ* = 1%	*ρ* = 2%	*ρ* = 5%	*ρ* = 10%	*ρ* = 20%	*ρ* = 30%
*B* = 10	44.0	25.8	9.5	5.0	1.2	1.0
*B* = 20	46.1	21.5	11.2	4.2	0.5	0.6
*B* = 30	43.3	31.7	7.8	4.6	1.1	1.0
*B* = 40	44.6	26.9	10.3	4.9	0.7	0.9

**Table 2:** Evaluation of the threshold selection for cutting long edges.

	2 × *median*	3 × *median*	4 × *median*	5 × *median*
*I_threshold_*	0.17	0.53	0.62	0.96

**Table 3:** Comparison of the clustering accuracy of the sum of squared error (SSE) between (parallel subsampling-based clustering) PARSUC(KM) and conventional K-means.

	500 × 500	1000 × 1000	2000 × 2000	3000 × 3000	4000 × 4000
PARSUC(KM)	2.11 × 10^8^	7.6 × 10^8^	1.35 × 10^9^	5.61 × 10^9^	0.23 × 10^10^
K-means	2.19 × 10^8^	7.72 × 10^8^	1.96 × 10^9^	6.33 × 10^9^	0.96 × 10^10^

**Table 4:** Comparison of the clustering accuracy (SSE) between PARSUC(ISO) and conventional ISODATA.

	500 × 500	1000 × 1000	2000 × 2000	3000 × 3000	4000 × 4000
PARSUC(ISO)	2.51 × 10^8^	8.80 × 10^8^	2.06 × 10^9^	6.92 × 10^9^	0.93 × 10^10^
ISODATA	2.36 × 10^8^	8.84 × 10^8^	2.14 × 10^9^	7.76 × 10^9^	1.66 × 10^10^

**Table 5:** Comparison of the processing time (in minutes) between PARSUC and MapReduce-based parallel clustering (MPC).

Image Size	Nodes = 5		Nodes = 10		Nodes = 15		Nodes = 20	
	PARSUC	MPC	PARSUC	MPC	PARSUC	MPC	PARSUC	MPC
5000 × 5000	32.49	87.47	16.97	50.44	12.23	37.45	9.17	27.34
10,000 × 10,000	134.06	305.61	69.94	169.88	48.75	122.49	37.12	92.25
20,000 × 20,000	516.39	1088.78	265.08	577.16	184.08	407.16	146.18	323.37
